# Brain-Derived Neurotrophic Factor Enhances Calcium Regulatory Mechanisms in Human Airway Smooth Muscle

**DOI:** 10.1371/journal.pone.0044343

**Published:** 2012-08-29

**Authors:** Amard J. Abcejo, Venkatachalem Sathish, Dan F. Smelter, Bharathi Aravamudan, Michael A. Thompson, William R. Hartman, Christina M. Pabelick, Y. S. Prakash

**Affiliations:** 1 Department of Anesthesiology, Mayo Clinic, Rochester, Minnesota, United States of America; 2 Department of Physiology and Biomedical Engineering, Mayo Clinic, Rochester, Minnesota, United States of America; Vanderbilt University Medical Center, United States of America

## Abstract

Neurotrophins (NTs), which play an integral role in neuronal development and function, have been found in non-neuronal tissue (including lung), but their role is still under investigation. Recent reports show that NTs such as brain-derived neurotrophic factor (BDNF) as well as NT receptors are expressed in human airway smooth muscle (ASM). However, their function is still under investigation. We hypothesized that NTs regulate ASM intracellular Ca^2+^ ([Ca^2+^]_i_) by altered expression of Ca^2+^ regulatory proteins. Human ASM cells isolated from lung samples incidental to patient surgery were incubated for 24 h (overnight) in medium (control) or 1 nM BDNF in the presence vs. absence of inhibitors of signaling cascades (MAP kinases; PI3/Akt; NFκB). Measurement of [Ca^2+^]_i_ responses to acetylcholine (ACh) and histamine using the Ca^2+^ indicator fluo-4 showed significantly greater responses following BDNF exposure: effects that were blunted by pathway inhibitors. Western analysis of whole cell lysates showed significantly higher expression of CD38, Orai1, STIM1, IP_3_ and RyR receptors, and SERCA following BDNF exposure, effects inhibited by inhibitors of the above cascades. The functional significance of BDNF effects were verified by siRNA or pharmacological inhibition of proteins that were altered by this NT. Overall, these data demonstrate that NTs activate signaling pathways in human ASM that lead to enhanced [Ca^2+^]_i_ responses via increased regulatory protein expression, thus enhancing airway contractility.

## Introduction

Neurotrophins (NTs) are growth factors primarily described in the nervous system where they play an integral role in neuronal development and function [1]–[3]. NTs such as brain derived neurotrophic factor (BDNF) function via both high affinity tropomyosin related kinase (TrkB in the case of BDNF) and low-affinity pan-neurotrophin (p75NTR) receptors to activate several intracellular signaling cascades including phospholipase C (PLC), phosphatidylinositol 3 kinase (PI3K), mitogen activated protein kinases (MAPK) and nuclear factor kappa-light chain-enhancer of activated B cells (NFκB) [4]–[6]. In this regard, studies in neuronal systems have reported both acute, non-genomic effects of NTs such as enhanced intracellular Ca^2+^ ([Ca^2+^]_i_) and synaptic transmission [7], [8], as well as genomic effects over longer time scales involving altered expression of genes and proteins [9], [10].

In addition to their well-recognized role in the nervous system, there is now increasing evidence that NTs and their receptors are expressed in a range of non-neuronal tissues including the lung [11]–[16]. For example, we recently demonstrated that BDNF, TrkB and p75NTR are all expressed by human airway smooth muscle (ASM) [13], [14], that acute exposure to BDNF enhances [Ca^2+^]_i_ responses to agonist [14] and potentiates the effects of pro-inflammatory cytokines such as tumor necrosis factor (TNFα) [13]. While these data suggest at least a non-genomic role for BDNF in the airway, as a growth factor that is released by several airway cells including epithelium, ASM, immune cells, and nerves [12], [17]–[19], BDNF presumably has longer term, genomic effects on cells of the airway. The relevance of such effects lies in the recent recognition that the levels of circulating and local BDNF, as well as receptor expression are increased in asthma [12], [17]. Furthermore, a role for BDNF in airway inflammation, remodeling and hyperreactivity has been suggested based on animal studies [20], [21]. However, the underlying mechanisms are still under investigation.

Diseases such as asthma are characterized by both enhanced airway contractility as well as remodeling which in turn may involve ASM cell proliferation. We recently demonstrated that in human ASM, prolonged BDNF exposure enhances cell proliferation [22], consistent with its role as a growth factor. Enhanced contractility may result from exaggerated responses to agonist, and here BDNF may act non-genomically to potentiate such responses [13]. Additionally, upregulation of mechanisms that normally regulate [Ca^2+^]_i_ and force in ASM also contribute to overall increases in contractility. We hypothesized that as a growth factor known to promote protein expression in the nervous system, prolonged BDNF exposure enhances expression and function of [Ca^2+^]_i_ and force regulatory components in ASM. We tested this hypothesis using primary human ASM cells and determined the role of signaling mechanisms most commonly associated with BDNF.

**Figure 1:**
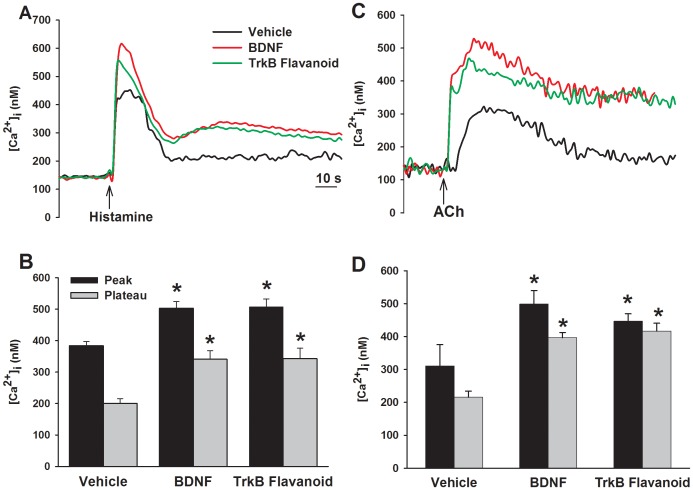
Effect of prolonged exposure to brain-derived neurotrophic factor (BDNF) on intracellular Ca^2+^ ([Ca^2+^]_i_) responses to agonist stimulation in human airway smooth muscle (ASM) cells. Figure shows representative tracings (A, C) and summaries of peak and plateau [Ca^2+^]_i_ responses (B, D) of ASM cells to 10 µM histamine (A, B) and 1 µM ACh (C, D) following 24 h exposure to vehicle (control), 1 nM BDNF, or 500 nM of the novel agonist 7,8-dihydroxyflavone which specifically activates the high-affinity BDNF receptor tropomyosin related kinase B (TrkB). The enhancing effects of BDNF and 7,8-dihydroxyflavone were comparable, suggesting that BDNF acts via TrkB in mediating effects on [Ca^2+^]_i_. Values are means ± SE (n = 4 patients). *Significant difference from control (p<0.05).

## Materials and Methods

### Isolation of Human ASM Cells

The techniques for isolating human ASM cells from lung samples incidental to patient surgery have been previously described [13], [14]. Pathologically normal areas of 3rd to 6th generation bronchi were dissected from lung samples of patients undergoing pneumenectomies or lobectomies for focal disease (de-identified samples considered surgical waste following clinical diagnoses; approved by Mayo’s Institutional Review Board and considered not Human Subjects Research. Accordingly, patient consent was waived). The IRB-approved protocols involved initial review of patient histories, to allow exclusion of smokers, asthmatics or patients with COPD, since we did not want to confound the effect of variable chronic inflammation and/or smoke exposure on the parameters studied. Following this step, samples were completely de-identified for storage and subsequent usage. In this study, we used airways from both males (3) and females (3).

Samples were placed in cold Hanks’ balanced salt solution (HBSS, Invitrogen, Carlsbad, CA) supplemented with 10 mM HEPES and 2 mM Ca^2+^. ASM layer was removed, minced and enzymatically dissociated using papain and collagenase with ovomucoid/albumin separation (Worthington Biochemical, Lakewood, NJ). Cell pellets were re-suspended and seeded into 75 cm^2^ sterile tissue culture flasks, 100 mm Petri dishes or 96-well clear bottom plates, and maintained under usual culture conditions in phenol red-free DMEM/F-12 (Invitrogen) supplemented with 10% FBS. Prior to experimentation, cells were serum starved for 48 h. Experiments were limited to passages 1–3 of subculture in order to ensure maintenance of ASM phenotype, which was periodically assessed by Western analysis for smooth muscle actin and myosin, and agonist receptors.

**Figure 2:**
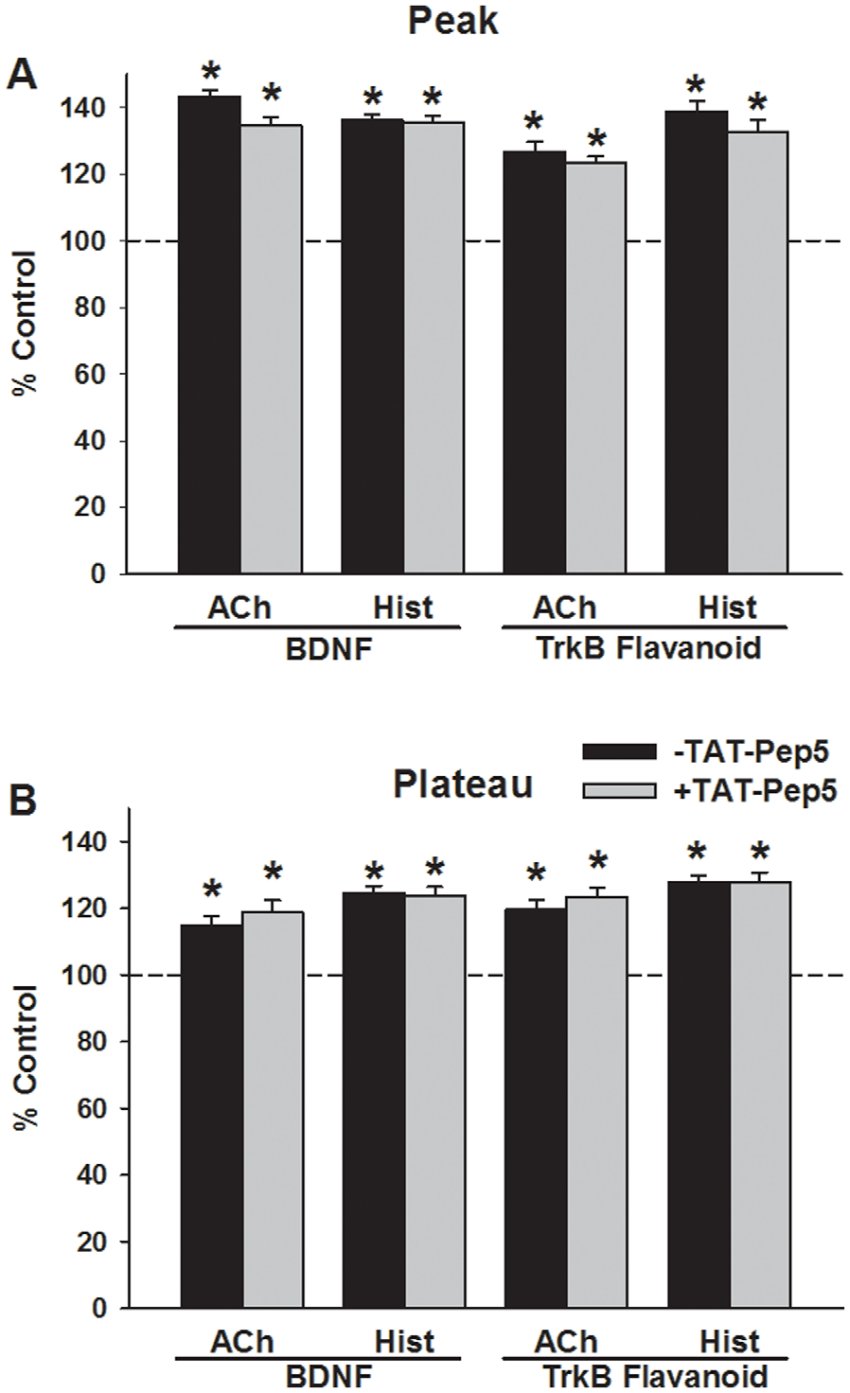
Role of the low-affinity neurotrophin receptor p75NTR in BDNF enhancement of [Ca^2+^]_i_ responses in human ASM cells. Pre-exposure of ASM cells to 1 µM TAT-pep5, a peptide that blocks p75NTR, had no significant effect on subsequent BDNF enhancement of [Ca^2+^]_i_ responses to ACh or histamine (A, B). TAT-pep5 also did not affect 7,8-dihydroxyflavone enhancement of [Ca^2+^]_i_. These data support the idea that BDNF acts via TrkB in enhancing [Ca^2+^]_i_. Values are means ± SE (n = 4 patients). *Significant difference from control (p<0.05).

### Cell Exposures

Human ASM cells were incubated for 24 h at 37°C in regular growth media (control), or 1 nM recombinant human BDNF (#248BD, R&D Systems, Minneapolis, MN) reconstituted in growth media. The BDNF concentration was selected based on our previous data showing acute effects on [Ca^2+^]_i_ [14] as well as pilot studies in which we found that 100 pM BDNF did not significantly affect [Ca^2+^]_i_ regulation, whereas 10 nM or higher concentration resulted in significant cell death with prolonged exposure. To determine the signaling mechanisms by which BDNF could affect ASM, additional sets of cells were incubated overnight in 1 nM BDNF in the presence of inhibitors of signaling pathways associated with BDNF: MAP kinase (PD98059, 10 µM, #9900, Cell Signaling, Danvers, MA), PI3/Akt (Wortmannin, 50 nM, #W1628, Sigma, St. Louis, MO) and NFκB (SN50, 20 µM, #481480, EMD Millipore, Billerica, MA) [22]. Other inhibitors of signaling pathways associated with BDNF included: Akt Inhibitor XII (500 nM, #124030, EMD Millipore), extracellular-regulated kinase (ERK) Activation Inhibitor Peptide I (2.5 µM, #328000, Sigma), Raf1 Kinase Inhibitor I (10 nM, #553008, EMD Millipore), RSK Inhibitor (100 nM, #559286, EMD Millipore), and IKK Inhibitor III (10 µM, #401480, EMD Millipore). To determine the role of the high-affinity receptor TrkB, ASM cells were also incubated overnight in 500 nM 7,8-dihydroxyflavone (#3826, Tocris, Minneapolis, MN), a newly identified, flavanoid TrkB-specific agonist [23]–[25]. To determine the role of the low-affinity receptor p75NTR, the specific inhibitor TAT-Pep5 (100 nM, #506181, EMD Millipore) was used.

**Figure 3:**
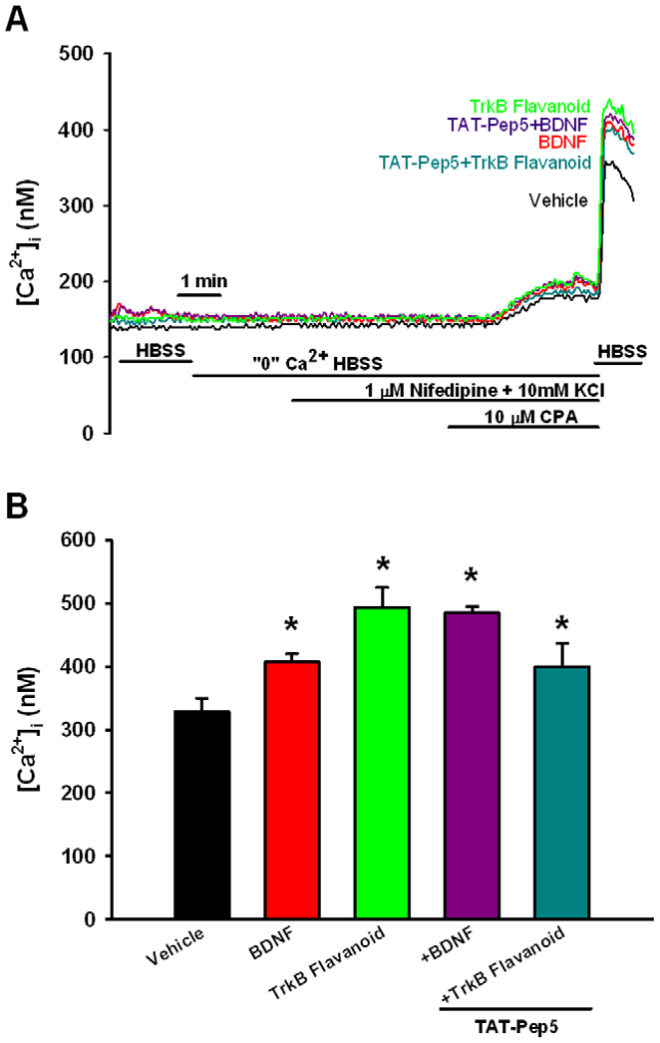
Effect of prolonged BDNF exposure on store-operated Ca^2+^ entry (SOCE) in human ASM cells. SOCE was evaluated by depleting sarcoplasmic reticulum stores in the absence of extracellular Ca^2+^, and then rapidly re-introducing extracellular Ca^2+^ in the continued presence of CPA [26]–[28] (A). Compared to vehicle controls, SOCE was significantly greater in cells exposed overnight to BDNF or to 7,8-Dihydroxyflavone. However, TAT-pep5 had no significant effect on either BDNF or 7,8-Dihydroxyflavone enhancement of SOCE (B). Values are means ± SE (n = 4 patients). *Significant difference from control (p<0.05).

### Ca^2+^ Measurements

ASM cells were incubated in 5 µM fluo-4/AM (#F14217, Invitrogen) for 60 min at room temperature and visualized using a 96-well fluorescence imaging plate reader with robotic pipetting capabilities (FlexStation 3, Molecular Devices, Sunnyvale, CA). Cells were initially perfused with HBSS (2.5 mM Ca^2+^ at room temperature), and baseline fluorescence levels established. Fluo-4 was excited at 495 nm and fluorescence emissions collected separately at 510 nm. [Ca^2+^]_i_ was quantified from fluo-4 levels using previously described empirical calibration procedures [14].

For agonist responses, ASM cells exposed to medium alone (control), or 1 nM BDNF in the presence or absence of inhibitors of signaling pathways were initially perfused with HBSS (2.5 mM Ca^2+^ at room temperature) for 30 s, and then exposed to 1 µM ACh or 10 µM histamine in HBSS to elicit [Ca^2+^]_i_ responses. The baseline, peak and plateau values of these responses were measured.

**Figure 4:**
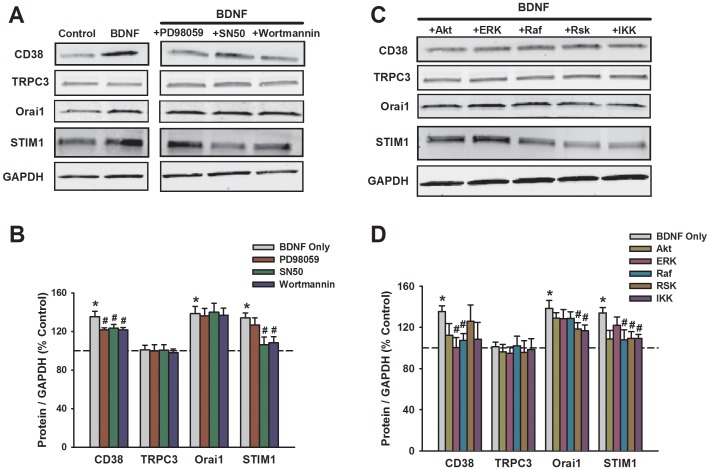
Effect of prolonged BDNF exposure on expression of membrane-associated Ca^2+^ regulatory proteins in human ASM cells. Western blot analysis demonstrated increased expression of the ectoenzyme CD38, and the Ca^2+^ influx regulatory components Orai1 and STIM1, but not TRPC3 (A, B). Inhibition of MAP kinases with PD98059, PI3/Akt with Wortmannin, or NFκB with SN50 had differential blunting effects on BDNF enhancement of CD38 vs. STIM1 vs. Orai1 (A, B; see text for detailed description). Consistent with these results, inhibition of signaling intermediates (see Methods for details) such as ERK, Raf, RSK and IKK blunted the effects of BDNF on CD38, while effects on STIM1 were more affected by IKK inhibition (C, D). In contrast, inhibition of Akt was generally less effective. Protein expression was normalized to GAPDH. *Significant difference from control; #Significant effect of inhibitor. Values are means ± SE (n = 4 patients).

For examining store-operated Ca^2+^ entry (SOCE), a major [Ca^2+^]_i_ regulatory mechanism in ASM [26]–[28], we used previously described protocols [26]. Briefly, ASM cells were washed with HBSS and extracellular Ca^2+^ removed by perfusion with zero-Ca^2+^ HBSS (5 mM EGTA). Cells were then exposed to 1 µM nifedipine and 10 mM KCl in the continued absence of extracellular Ca^2+^ and the sarcoplasmic reticulum passively depleted by 10 µM cyclopiazonic acid (CPA), an inhibitor of the sarco(endo)plasmic reticulum Ca^2+^-ATPase (SERCA). The intent of adding nifedipine and KCl was to prevent unwanted Ca^2+^ influx via L-type channels, and to clamp the membrane potential to avoid Ca^2+^ entry via other voltage-sensitive channels [26]. Following sarcoplasmic reticulum depletion, 2.5 mM extracellular Ca^2+^ was rapidly reintroduced in the continued presence of CPA and the observed [Ca^2+^]_i_ elevation relative to the levels in 0 Ca^2+^ and CPA measured as an index of SOCE.

### siRNA Transfections

The techniques for siRNA transfection in human ASM cells has been previously described [22]. Briefly, human ASM cells were grown on 8 well Lab-teks or 100 mm plates to approximately 50% confluence. Negative control (#4611) or protein-specific siRNAs (CD38, 216908; Orai1, 119606; Stromal interaction molecule 1 (STIM1), 4392421; Invitrogen; Transient receptor potential cation channel 3 (TRPC3), J-006509-07; Thermo Scientific; 20 nM each) were permitted to form complexes with Lipofectamine™ 2000 per manufacturer’s instructions. Culture media was removed and replaced with fresh serum free DMEM/F12 lacking antibiotics. siRNA and Lipofectamine complexes were then added for 6 h followed by addition of DMEM/F12 containing 10% FBS. After 24 h, transfection medium was removed and replaced with fresh DMEM/F12 containing 10% FBS for an additional 24 h. Finally, serum free DMEM/F12 was used for an additional 48 h, following which experimental protocols were performed. Specific protein suppression was confirmed by Western analysis.

**Figure 5:**
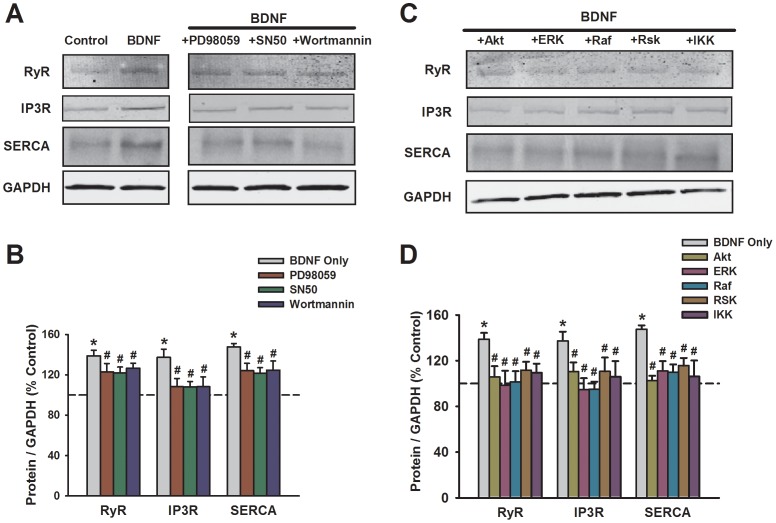
Effect of prolonged BDNF exposure on expression of sarcoplasmic reticulum Ca^2+^ regulatory proteins in human ASM cells. Western blot analysis demonstrated increased expression of both Ca^2+^ release mechanisms IP_3_R and RyR channels, as well as the Ca^2+^ reuptake protein SERCA (A, B). Compared to membrane regulatory proteins (Figure 4), inhibition of MAP kinases, PI3/Akt or NFκB (A, B) or their signaling intermediates (C, D) were equally effective in blunting BDNF enhancement of Ca^2+^ regulatory proteins. Protein expression was normalized to GAPDH. *Significant difference from control; #Significant effect of inhibitor. Values are means ± SE (n = 4 patients).

### Western Blot Analysis

Proteins were separated by SDS-PAGE (Criterion Gel System; Bio-Rad, Hercules, CA; either 7.5%, 10%, 15% or 4–15% gradient gels) for approximately one hour and then transferred to nitrocellulose membranes (Bio-Rad) for 7 min using Trans-Blot Turbo transfer system (Bio-Rad). 5% milk in TBS was used to block the membranes for 1 h. Membranes were then incubated overnight at 4°C with primary antibodies at 1 µg/ml concentration. Following three washes with TBS, proteins were detected using infrared range secondary antibodies developed for the Odyssey Li-Cor Imaging System (Lincoln, Nebraska). Antibodies used included: CD38 (Epitomics, 2935-1), TRPC3 (Alomone, ACC-016), Orai1 (Alomone, ACC-060), STIM1, (ABCAM, ab62031), ryanodine receptor (RyR, ABCAM, ab2868), inositol 1,4,5-trisphosphate receptor (IP_3_R, Cell Signaling, 3763), SERCA (ABCAM, ab2818), and GAPDH (Cell Signaling, 2118).

### Statistical Analysis

Bronchial samples from at least 4 patients were used to obtain ASM cells, with biochemistry and molecular biology protocols as well as [Ca^2+^]_i_ measurements being repeated a minimum of 3 times per patient. Not all Ca^2+^ measurement protocols were performed in each subculture of cells due to time constraints. Drug effects on [Ca^2+^]_i_ responses were compared across sets of cells (independent *t*-test) with Bonferroni correction for repeated comparisons. Statistical significance was established at p<0.05. All values are expressed as means ± SE.

## Results

### Effect of BDNF and 7, 8-Dihydroxyflavone on [Ca^2+^]_i_ Regulation

Exposure of control ASM cells to ACh or histamine produce characteristic [Ca^2+^]_i_ responses involving an initially higher peak followed by a decay to a plateau higher than baseline. Exposure to 1 nM BDNF for 24 h enhanced both peak and plateau [Ca^2+^]_i_ responses to ACh and histamine compared to vehicle controls (Figure 1; p<0.05 for BDNF effects on peak and plateau responses for either agonist). Exposure to the TrkB-agonist 7,8-Dihydroxyflavone only (no additional BDNF) for 24 h increased both peak and plateau [Ca^2+^]_i_ responses to agonists to levels comparable to that with BDNF (Figure 1; p<0.05). However, overnight exposure to 1 µM TAT-pep5, a peptide that blocks the low-affinity p75NTR, did not affect BDNF-induced or 7,8-Dihydroxyflavone induced enhancement of peak or plateau [Ca^2+^]_i_ responses to ACh or histamine (Figure 2).

**Figure 6:**
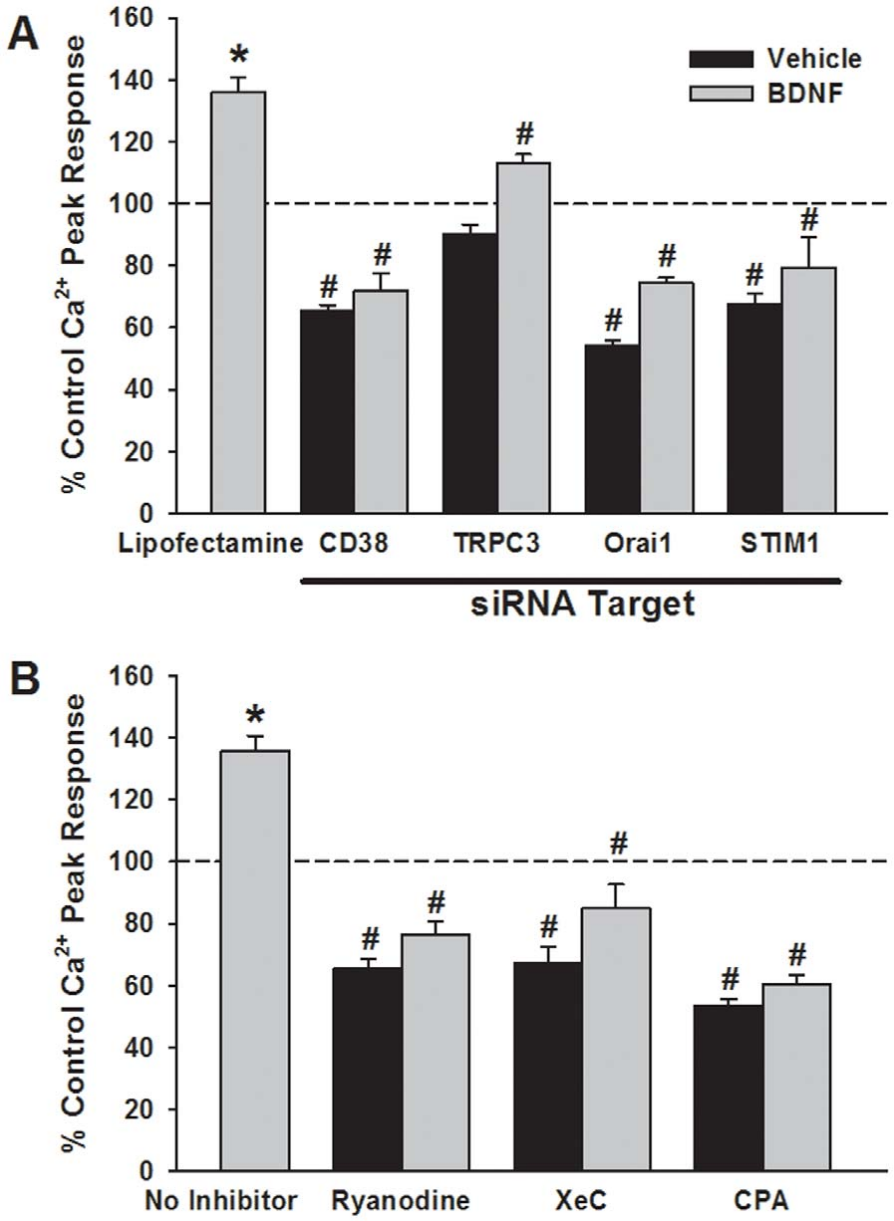
Functional consequence of BDNF-induced changes in Ca^2+^ regulatory protein expression. Small interference RNA (siRNA) inhibition of CD38, Orai1 or STIM1 expression substantially reduced peak [Ca^2+^]_i_ responses to histamine not only in cells exposed to vehicle only (i.e. no BDNF), but also in those exposed for 24 h to BDNF (A). Transfection agent (Lipofectamine) or nonsense siRNA had no effect on [Ca^2+^]_i_ responses in either group (not shown). In contrast, siRNA inhibition of TRPC3 had substantially less effect on BDNF enhancement of peak [Ca^2+^]_I_, suggesting that the other mechanisms were functionally linked to BDNF effects, and consistent with no change in TRPC3 expression. Similarly, in ASM cells exposed to BDNF for 24 h, inhibition of RyR channels (high concentration ryanodine) or IP_3_ channels (Xestospongin C, XeC) or of SERCA (with cyclopiazonic acid; CPA) significantly blunted BDNF enhancement of peak [Ca^2+^]_i_ responses to histamine (B). *Significant difference from control; #Significant effect of inhibitor. Values are means ± SE (n = 4 patients).

Store-operated Ca^2+^ entry was evaluated by depleting sarcoplasmic reticulum stores in the absence of extracellular Ca^2+^, and then rapidly re-introducing extracellular Ca^2+^ in the continued presence of CPA. Compared to vehicle controls, SOCE was significantly greater in cells exposed overnight to BDNF (Figure 3; p<0.05). Exposure to 7, 8-Dihydroxyflavone also produced comparable increase in SOCE (p<0.05), while TAT-pep5 had no significant effect on either BDNF or 7,8-Dihydroxyflavone enhancement of SOCE.

### Effect of BDNF on Ca^2+^ Regulatory Proteins

Western blot analysis of whole cell lysates from human ASM demonstrated complex effects of BDNF on Ca^2+^ regulatory proteins. BDNF increased expression of Ca^2+^ influx regulatory components Orai1 and STIM1 (Figure 4; p<0.05), as well as sarcoplasmic reticulum proteins SERCA (Ca^2+^ reuptake), IP_3_R and RyR channels (Ca^2+^ release) (Figure 5; p<0.05) and the second messenger regulating ectoenzyme CD38 [29] (Figure 4, p<0.05). However, TRPC3 expression was unchanged by BDNF exposure (Figure 4).

**Figure 7:**
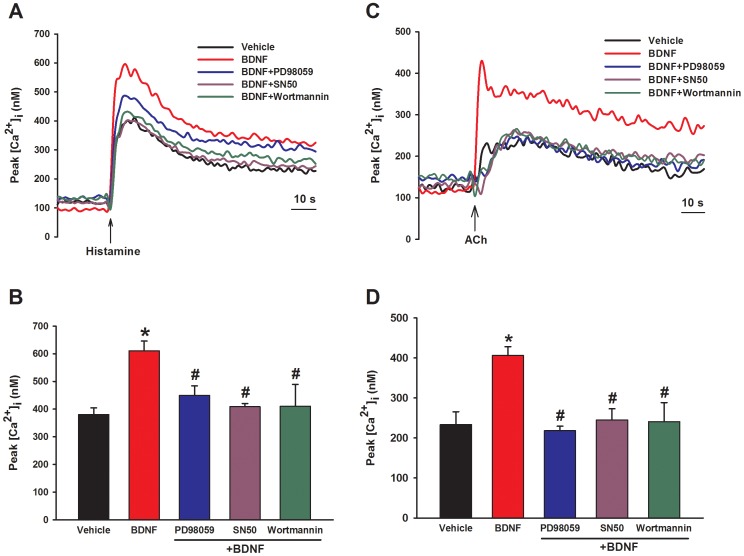
Effect of signaling cascade inhibitors on BDNF enhancement of peak [Ca^2+^]_i_ responses in human ASM cells. Overnight incubation of ASM cells with either PD98059 or SN50 in the presence of BDNF substantially blunted the enhanced peak [Ca^2+^]_i_ responses observed with BDNF only for both histamine (A, B) and ACh (C, D). In summary graphs (B, D), *Significant difference from control; #Significant effect of inhibitor. Values are means ± SE (n = 4 patients).

To determine the mechanisms by which BDNF affects Ca^2+^ regulatory protein expression, pharmacological inhibition of signaling pathways thought to be activated by BDNF was used. Inhibition of these pathways generally blunted the enhancing effects of BDNF on expression of all the proteins mentioned above (except TRPC3 obviously), but the contribution of specific pathways differed between the Ca^2+^ regulatory proteins. There was no significant effect of the MAP kinase inhibitor PD98059 (10 µM) on influx mechanisms: TRPC3, Orai1, or STIM1. However, MAP kinase inhibition blunted BDNF effects on SR Ca^2+^ regulatory proteins (Figures 4 vs. 5; p<0.05). Based on these results, we explored the effect of inhibiting specific signaling intermediates relevant to MAP kinases. As expected TRPC3 expression which was unaffected by BDNF, as also not influenced by any of the inhibitors. Enhanced expression of BDNF of CD38 was suppressed by pharmacological inhibition of ERK, Raf, and Rsk (Figure 4; p<0.05). Interestingly, even though PD98059 did not inhibit BDNF effect on STIM1 or Orai1, inhibition of ERK, Raf and/or Raf had small but significant blunting effect (Figure 4; p<0.05). In a similar vein, there were differential effects of PI3 vs. Akt inhibition on BDNF enhancement of CD38 or STIM1. Compared to these pathways, inhibition of NFκB or IKK consistently blunted BDNF effects on a number of proteins (Figure 4; p<0.05). Here, inhibition of multiple signaling pathways blunted BDNF effects on expression of intracellular Ca^2+^ regulatory proteins SERCA, IP3 receptor and RyR channels (Figure 5; p<0.05 for all inhibitors).

**Figure 8:**
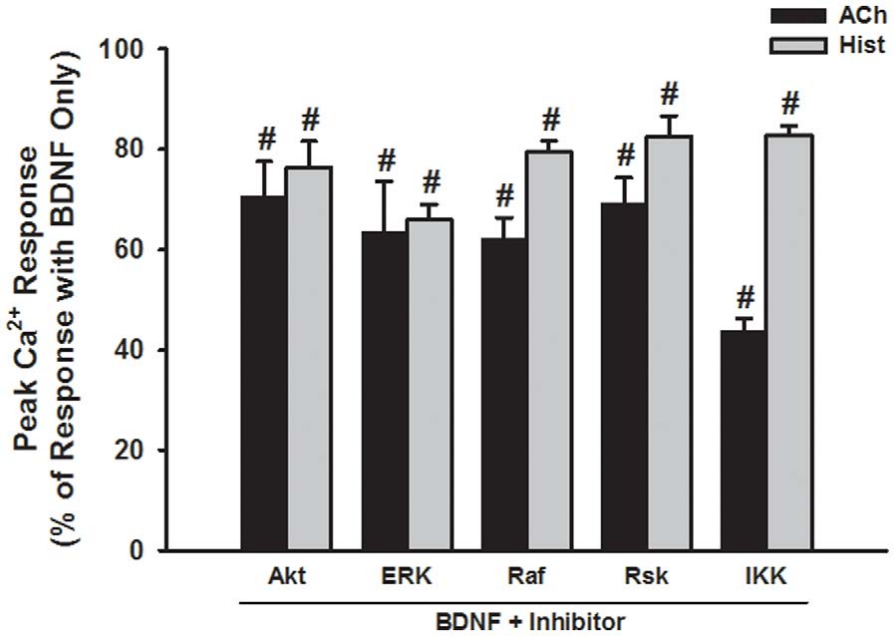
Effect of inhibiting signaling intermediates on BDNF enhancement of peak [Ca^2+^]_i_ responses in human ASM cells. Compared to the enhanced [Ca^2+^]_i_ response to ACh or histamine in BDNF-exposed cells, the presence of inhibitors for intermediates of MAP kinase, PI3/Akt or NFκB pathways resulted in peak [Ca^2+^]_i_ responses that were substantially blunted. #Significant effect of inhibitor. Values are means ± SE (n = 4 patients).

The functional consequence of enhanced Ca^2+^ regulatory protein expression by BDNF was determined by examining [Ca^2+^]_i_ responses when the relevant proteins were pharmacologically or genetically suppressed. Consistent with the pattern of protein expression, siRNA inhibition of CD38, Orai1 or STIM1 expression significantly blunted BDNF enhancement of peak [Ca^2+^]_i_ responses to histamine (Figure 6; p<0.05 for each siRNA). Transfection vehicle or nonsense siRNA were without effect (not shown). Inhibition of SERCA with 10 µM CPA, of RyR channels with 10 µM ryanodine (#559276, EMD Millipore), or of IP3 receptor channels with 500 nM Xestospongin C (XeC, #682160, EMD Millipore) all prevented enhancement of peak [Ca^2+^]_i_ responses to histamine by BDNF (Figure 6; p<0.05). Overnight incubation of ASM cells with either PD98059, SN50 or Wortmannin in the presence of BDNF substantially blunted the enhanced peak [Ca^2+^]_i_ responses observed with BDNF only (Figure 7; p<0.05 for either inhibitor). Consistent with these results, inhibition of signaling intermediates (as for protein expression) also prevented BDNF enhancement of peak [Ca^2+^]_i_ responses to both ACh and histamine (Figure 8; p<0.05 for each inhibitor).

## Discussion

The present study examined signaling mechanisms by which neurotrophins influence [Ca^2+^]_i_ in human ASM and found that prolonged exposure to BDNF activates multiple intracellular cascades leading to complex, enhancing effects on expression and function of Ca^2+^ regulatory proteins. Given previous studies demonstrating upregulation of airway BDNF in asthma and allergic diseases these data suggest that prolonged BDNF exposure can contribute to enhanced ASM contractility via genomic effects. Extensive studies in the nervous system have shown that secreted BDNF (cleaved extracellularly to an active form) specifically binds to high-affinity full-length TrkB receptors [30], [31], as well as the low-affinity pan-NT p75NTR receptor. These receptors can activate multiple signaling pathways including phospholipase Cγ, ERK, PI3K/Akt, and NFκB [30], [31], mechanisms that are well-recognized in ASM as being important for [Ca^2+^]_i_ regulation, cell proliferation and migration, and in mediating effects of pro-inflammatory cytokines such as TNFα and IL-13 in asthma [32], [33]. Thus, in combination with our previous data showing that acute BDNF enhances ASM [Ca^2+^]_i_ and force [13], [14], while prolonged exposure increases cell proliferation especially in the presence of inflammation [22], a major relevance of our study lies in the potential for NTs to influence all aspects of ASM relevant to airway diseases.

We and others have now established that NTs and their receptors are present in different lung components, with BDNF produced by airway epithelium, sensory innervation, ASM itself, and a host of immune cells (see [12], [17], [19] for review). Importantly, increased BDNF and receptor expression has been found in blood, tissue and lung lavages of patients with asthma and chronic bronchitis [12], [17], suggesting a role for BDNF in altered airway structure and function during inflammation. Importantly, previous data shows that ASM itself is both a potential source [14], [15] as well as a target [13], [14], [22] for BDNF, with effects being mediated via TrkB as well as p75NTR [13], [15]. In this regard, BDNF has acute, likely non-genomic effects of enhancing human ASM [Ca^2+^]_i_ and contractility [14], [34], [35], especially in the presence of inflammation [13]. The present study now shows that with prolonged BDNF exposure would further contribute to enhanced contractility by upregulation of proteins involved in [Ca^2+^]_i_ regulation.

The mechanisms by which NTs can influence airway structure or function are still under investigation. NTs can modulate neural influences, thus indirectly increasing airway contractility [36], [37]. However, as shown in previous studies, BDNF can also directly influence ASM [Ca^2+^]_i_ [14]. Here, expression of TrkB and p75NTR allows ASM to react to NTs derived from other sources as well. In ASM, [Ca^2+^]_i_ responses to bronchoconstrictor agonist involve sarcoplasmic reticulum Ca^2+^ release via IP_3_R [38] and RyR channels [39] as well as Ca^2+^ influx via voltage-gated channels [40], receptor-gated channels [41], [42] and SOCE in response to intracellular Ca^2+^ depletion [26], [43]. In neurons, BDNF primarily increases Ca^2+^ release from intracellular stores [44]–[46], mediated via TrkB, phospholipase C, IP_3_ production and IP_3_R channels [47]. We previously showed that BDNF acutely enhances [Ca^2+^]_i_ responses to agonists such as histamine that largely affect IP_3_R channels as well as to caffeine which opens RyR channels [14]. In pontine neurons of newborn rat, BDNF increases influx via TRPC3 channels [48], which are involved in SOCE of ASM as well [26]. Thus BDNF can enhance multiple [Ca^2+^]_i_ regulatory mechanisms. Our data now show that with prolonged exposure, such enhancement can be sustained long-term by increased expression of many of these proteins including IP_3_R and RyR channels. In addition BDNF also increases expression of Orai1, which has been recently recognized to be a key mediator (if not the channel) for SOCE [49]. These changes are reflected by the enhanced peak and plateau [Ca^2+^]_i_ values with agonist stimulation as well as SOCE.

In terms of [Ca^2+^]_i_ changes, the data showing comparable effects of BDNF and the newly-identified TrkB-specific agonist 7,8-dihydroxyflavone suggest a large role for TrkB signaling in ASM, with a likely small and insignificant role for p75NTR evidenced by the lack of effect of the p75NTR inhibitor TAT-pep5. In cell types other than ASM, BDNF activates a number of pathways including PI3/Akt, ERK1/2 and NFκB. In a recent study [22], we showed that activation of ERK1/2 was essential for BDNF-induced enhancement of ASM cell proliferation, thus suggesting involvement of CREB-mediated gene regulation [50]–[52]. This signaling cascade also appears to be important in BDNF-induced enhancement of [Ca^2+^]_i_ as well as Ca^2+^ regulatory protein expression in ASM, evidenced in this study by the effects of different pharmacological inhibitors for various signaling intermediates along this pathway. BDNF activation of ERK1/2 appears to involve TrkB as previously shown using TrkB siRNA [22]. Activation of NFκB also appears to be important in mediating BDNF effects on cellular proliferation [22], as well as [Ca^2+^]_i_ and protein expression as shown in this study using pharmacological inhibition of both IKK and NFκB. In this regard, the lack of effect of TAT-pep5 on BDNF enhancement of [Ca^2+^]_i_ is interesting since NFκB activation is typically associated with p75NTR signaling. However, the involvement of PI3/Akt in BDNF effects suggests an alternative route for NFκB activation which may be mediated via TrkB.

While the data in this study using pharmacological inhibitors suggest a role for all of MAP kinase, PI3/Akt and NFκB in mediating the effect of BDNF on expression of [Ca^2+^]_i_ regulatory proteins, it is interesting that their contributions are complex and appear to be mechanism specific. For example, MAP kinases appear to consistently affecting the intracellular Ca^2+^ regulatory proteins such as SERCA and release channels, but not influx mechanisms in general. On the other hand, NFκB appears to have a broad and consistent effect on both influx and intracellular Ca^2+^ mechanisms. The relative contribution of PI3 vs. Akt is even more complex, with both involved for some proteins but not others. These data only highlight the complexity of BDNF signaling in any cell type, and emphasize the need to examine the underlying mechanisms in every cell type of interest rather than extrapolating from the nervous system or other cell types. Indeed, we propose that the differential contribution of different signaling pathways in mediating BDNF effects allows BDNF to have pleiotropic effects on inflammation and other insults that also use these signaling cascades. Accordingly, in diseases such as asthma, BDNF has the potential to enhance one cascade vs. the other to enhance [Ca^2+^]_i_ or cell proliferation as we have previously shown. [22].

The present study demonstrates a functional relevance for the altered Ca^2+^ regulatory expression observed with BDNF, as well as the role of the above signaling cascades. Given the importance of the plasma membrane vs. intracellular regulatory mechanisms for [Ca^2+^]_i_ regulation in ASM, the effects of BDNF on CD38 are particularly important. This interesting ectoenzyme is a key molecule for the cyclic ADP ribose-RyR channel second messenger-based Ca^2+^ regulatory pathway in ASM. CD38 expression is known to be regulated by cascades such as MAP kinases and NFκB, and is considered a key mechanism in mediating the effects of inflammation [29]. Accordingly, BDNF-induced expression and function of CD38 has the potential to substantially influence airway reactivity and its dysfunction. Whether a similar relationship between BDNF and other [Ca^2+^]_i_ regulatory mechanisms holds in terms of signaling cascades is not yet known. However, the differential effects of inhibiting MAP kinase vs. PI3/Akt vs. NFκB on BDNF enhancement of influx proteins (STIM1, Orai1) vs. intracellular proteins (SERCA or RyR) suggest that BDNF can modulate the relative contribution of these Ca^2+^ pathways in a complex fashion. This pleiotropic effect will facilitate nuanced influences of BDNF on [Ca^2+^]_i_ regulation via influx vs. intracellular release/reuptake in the setting of inflammation where certain mechanisms may be upregulated, such as influx [26]–[28], while others such as SERCA may decrease.

In conclusion, the present study shows that BDNF upregulates a number of mechanisms important in airway contractility which, in combination with enhancement of ASM cell proliferation and cytokine effects can substantially influence airway hyperresponsiveness. Further in vivo research is required to determine whether interactions between BDNF and inflammation is enhanced in asthmatic airways, setting the stage to investigate the potential for interference with BDNF signaling as a therapeutic avenue.
